# Psychological impact of fertility preservation techniques in women with gynaecological cancer

**DOI:** 10.3332/ecancer.2017.ed62

**Published:** 2017-02-08

**Authors:** Antonio Simone Laganà, Valentina Lucia La Rosa, Agnese Maria Chiara Rapisarda, Alessio Platania, Salvatore Giovanni Vitale

**Affiliations:** 1 Unit of Gynaecology and Obstetrics, Department of Human Pathology in Adulthood and Childhood “G Barresi”, University of Messina, Messina, 98125, Italy; 2 Unit of Psychodiagnostics and Clinical Psychology, University of Catania, Catania, 95124, Italy; 3 Department of General Surgery and Medical Surgical Specialties, University of Catania, Catania, 95124, Italy; 4 Department of Health Services and Epidemiological Observatory, Palermo, 90121, Italy

**Keywords:** female, fertility preservation, reproduction, quality of life

## Abstract

Gynaecological cancer is a very stressful experience for women and treatments can compromise fertility and reproductive capacity. Fertility preservation techniques in women with gynaecological cancer can play an important role in improving the quality of life of these patients but, in many cases, the information about this type of treatment is not adequate. It is important to further investigate this topic in order to reduce the impact of gynaecological cancer on the quality of life of survivors as much as possible.

Gynaecological cancer affects around 17% of women worldwide [1]. The most common types are endometrial cancer followed by ovarian, cervical, and vulvar cancer [1, 2], although other rare malignancies may occur [3]. Endometrial cancer is the most common gynaecological malignancy in Western countries [4]. Surgery is the primary treatment for most patients while adjuvant radiation therapy or chemotherapy is recommended in more advanced stages of disease [5–7]. Ovarian cancer is the most lethal form of gynaecological cancer and it is generally diagnosed in an advanced stage [1]. Treatment consists of a combination of extensive surgery and chemotherapy, and it is often associated with high incidence of adverse events [8–10] and recurrence [11, 12]. Cervical cancer is often diagnosed during the reproductive years [1]. Treatment may consist of a combination of surgery and radiation therapy [13]. Vulvar cancer accounts for 3–5 % of all gynaecological cancers [1]. It usually occurs in older women but it can also appear in younger women [1]. Treatment is surgical with or without adjuvant radiotherapy [14].

The diagnosis of a gynaecological cancer is a very stressful experience for women with a significant impact on their psychological, sexual and social functioning [1, 2, 15, 16]. Women with gynaecological cancer suffer from depression, anxiety, suicidal ideation, feelings of anger and shame, lower self-esteem and a poor quality of life (QoL) [1, 2, 15–19]. Moreover, treatments for gynaecological cancer often lead to physical and sexual changes that impair female identity and sexual functioning [1, 2, 15, 16]. Thanks to the most recent advances in cancer therapy, the number of long-term gynaecological cancer survivors has increased and a primary objective with these patients is to improve their quality of life and psychological well-being. Gynaecological cancer often affects women in childbearing age and treatments can compromise fertility and reproductive capacity. Young women with a gynaecological cancer often experience psychological distress with the loss of menstrual function and the loss of fertility. In particular, psychological distress related to infertility is more significant in women who have not yet started their families and would still like to do so [20–22].

A cancer survivor may assign a high value to parenthood because a child is a symbol of life that defeats death and the opportunity for women to give birth to something beautiful [23–26]. Consequently, fertility preservation techniques in women with gynaecological cancer can play an important role in improving quality of life of these patients. Despite the fact that gynaecological cancers often require radical surgery which deprives the patient of her fertility, the development of fertility-sparing surgery (FSS) has radically changed the scenario of fertility preservation: regarding cervical cancer, radical trachelectomy with lymph node assessment became the standard of care for selected women with lesions <2 cm who desire fertility preservation [27]; in addition, for patients ineligible for FSS or who require adjuvant radiation therapy, current options include ovarian transposition and cryopreservation of oocytes or embryos [28]. Regarding endometrial cancer, new evidence regarding the possibility of fertility-sparing treatment in young patients with early stage (IA, G1 and 2) endometrioid endometrial cancer treated by combined hysteroscopic resection and progestin therapy changed the perspective of the disease [29].

A recent systematic review suggests also that FSS is feasible and safe in early-stage epithelial ovarian cancer (stage IA and IC grade 1 and 2 disease and stage IC1 according to the new FIGO staging system) [30]. In all the cases in which it is not possible to perform fertility-sparing treatments, radical surgery treatment (hysterectomy, bilateral ovariectomy) deprives patients of the possibility to have offspring. In addition, (neo)adjuvant treatments (radio and/or chemotherapy) may also play a detrimental role on fertility and must be tailored to the patient’s individual characteristics [31]. The most effective fertility preservation strategy is embryo cryopreservation which requires in vitro fertilization and a male partner [32]. Oocyte banking and GnRH analog treatment are more problematic and do not preserve fertility in all patients. Currently, cryopreservation of ovarian tissue appears to be a very promising fertility preservation technique. It is an easy, fast and less expensive technique that has excellent prospects of fertility preservation [32, 33].

Physicians don’t always discuss fertility preservation with cancer patients of childbearing age and this may be due to their insufficient knowledge regarding these techniques and lack of specific communication strategies [21, 34]. Many factors may interfere with the communication between patient and physician concerning fertility preservation: physician knowledge of fertility preservation techniques and their efficacy, distress and anxiety of the patient, and financial problems may adversely affect decision-making process about fertility preservation [35]. This process is complex and many factors can come into play. For example, cognitive aspects, such as biases and heuristics, may make decision-making about fertility preservation more difficult and less aware [36]. In these cases, decision support interventions or decision aids are appropriate to facilitate and support the decision-making process, reducing the possible biases [36]. Decision aids increase knowledge and reduce decisional conflict without increasing anxiety. They provide information about cancer and female fertility, and discuss the different available fertility options and the advantages and disadvantages of each one [37]. Also the P5 approach may be very useful to improve decision-making about fertility preservation and to transform patients into active decision-makers in the treatment process [38].

Therefore it is important to provide complete information about fertility-preservation options, in order to take into account the patient’s individual background and to consider the patient’s emotional and psychological needs. Adequate counseling about fertility preservation techniques improves the quality of life of women who survive after gynaecological cancer [39, 40]. A multidisciplinary approach is important and psychologists should be involved in a team approach to cancer care. Indeed, reproductive counseling aims not only to choose the most appropriate fertility preservation technique according to the prognosis and the risk of infertility related to cancer treatments, but also to assess the real desire of woman to become mothers. Moreover, considering the impact of gynaecological cancer on the psychological well-being of women affected and the complexity of the decisions surrounding fertility preservation, psychosocial counseling may be useful to discuss the psychological problems and stressors associated with cancer and fertility preservation [41–43].

## Conclusions

Despite the importance of this topic, studies on the psychological and emotive effects of fertility preservation techniques are still scarce. For this reason, we strongly suggest the further investigation of this topic in order to reduce as much as possible the impact of gynaecological cancer on the quality of life of survivors.

## Conflicts of interest

The authors declare that they have no conflict of interest.

## Authors’ contributions

Antonio Simone Laganà conceived the manuscript; Valentina Lucia La Rosa wrote the manuscript; Agnese Maria Chiara Rapisarda and Alessio Platania drafted the article and revised it for critically important intellectual content; Salvatore Giovanni Vitale gave the final approval of the version to be published.
